# Adoption of Machine Learning Systems for Medical Diagnostics in Clinics: Qualitative Interview Study

**DOI:** 10.2196/29301

**Published:** 2021-10-15

**Authors:** Luisa Pumplun, Mariska Fecho, Nihal Wahl, Felix Peters, Peter Buxmann

**Affiliations:** 1 Software & Digital Business Group Technical University of Darmstadt Darmstadt Germany

**Keywords:** machine learning, clinics, diagnostics, adoption, maturity model

## Abstract

**Background:**

Recently, machine learning (ML) has been transforming our daily lives by enabling intelligent voice assistants, personalized support for purchase decisions, and efficient credit card fraud detection. In addition to its everyday applications, ML holds the potential to improve medicine as well, especially with regard to diagnostics in clinics. In a world characterized by population growth, demographic change, and the global COVID-19 pandemic, ML systems offer the opportunity to make diagnostics more effective and efficient, leading to a high interest of clinics in such systems. However, despite the high potential of ML, only a few ML systems have been deployed in clinics yet, as their adoption process differs significantly from the integration of prior health information technologies given the specific characteristics of ML.

**Objective:**

This study aims to explore the factors that influence the adoption process of ML systems for medical diagnostics in clinics to foster the adoption of these systems in clinics. Furthermore, this study provides insight into how these factors can be used to determine the ML maturity score of clinics, which can be applied by practitioners to measure the clinic status quo in the adoption process of ML systems.

**Methods:**

To gain more insight into the adoption process of ML systems for medical diagnostics in clinics, we conducted a qualitative study by interviewing 22 selected medical experts from clinics and their suppliers with profound knowledge in the field of ML. We used a semistructured interview guideline, asked open-ended questions, and transcribed the interviews verbatim. To analyze the transcripts, we first used a content analysis approach based on the health care–specific framework of nonadoption, abandonment, scale-up, spread, and sustainability. Then, we drew on the results of the content analysis to create a maturity model for ML adoption in clinics according to an established development process.

**Results:**

With the help of the interviews, we were able to identify 13 ML-specific factors that influence the adoption process of ML systems in clinics. We categorized these factors according to 7 domains that form a holistic ML adoption framework for clinics. In addition, we created an applicable maturity model that could help practitioners assess their current state in the ML adoption process.

**Conclusions:**

Many clinics still face major problems in adopting ML systems for medical diagnostics; thus, they do not benefit from the potential of these systems. Therefore, both the ML adoption framework and the maturity model for ML systems in clinics can not only guide future research that seeks to explore the promises and challenges associated with ML systems in a medical setting but also be a practical reference point for clinicians.

## Introduction

### Machine Learning Systems for Medical Diagnostics

The ongoing digitalization is influencing the everyday activities of almost every individual, both in their private and professional lives. This transformation is particularly evident in health care, where the integration of health information technologies (HITs), such as electronic health records or clinical decision support systems, enables significant improvements in processes such as emergency medical care, diagnostics, and therapy [1-3]. However, the integration of HITs is not a panacea but leads to major challenges in clinics as, fueled by these technologies, physicians have to handle an ever-growing volume of patient data and complexity of interacting systems [4]. Moreover, societal problems further complicate the provision of health services to the population, as age-related diseases are on the rise because of demographic shifts and global pandemics such as the COVID-19 crisis are overburdening clinics, pushing medical personnel to the limits of their capacity [5,6].

Artificial intelligence (AI) as the “science and engineering of making intelligent machines, especially intelligent computer programs” [7] could help relieve this burden on physicians as AI is capable of solving tasks previously reserved for human intelligence [8]. In particular, machine learning (ML), as a subfield of AI, is currently one of the fastest growing technological approaches, opening up a wide range of possibilities for medicine [9,10]. Therefore, in the remainder of this research work, we focus on ML systems, that is, information systems (IS) that learn to perform certain tasks autonomously through experience without receiving explicit human instructions. Instead, ML systems use algorithms to search large amounts of data for patterns to create their own rules and strategies on how to deal with a particular problem. The identified rules can then be applied to solve a task [9,11-13]. ML systems can be particularly useful in solving problems for which the rules are difficult to derive and express. This is the case, for example, in image recognition; for instance, how can the image of a cat be explained in terms of pixels, what shapes of ears are allowed, and how can they be recognized in a picture [13]. From the prediction of patient admissions in clinics to therapy support, ML systems can help solve various problems in medicine [10,14]. However, one application area of particular value to researchers and practitioners in which ML systems could have a major impact on the overall well-being of the population is medical diagnostics [15,16]. In this context, ML systems can help identify patterns in medical data (eg, in medical scans, pathology slides, electrocardiograms, and written diagnoses) and sort possible conditions according to their likelihood [17,18]. A distinction can be made between ML serving to take over entire areas of responsibility from physicians and supporting them in their decision-making process. In the near future, ML systems will mainly be used as intelligent decision support rather than to automate medical diagnostics fully [10,17,19,20]. Thus, current cases in research and practice show that an increasing number of such assistive ML systems are presently finding their way into medical workflows. For example, ML systems are being developed, refined, and deployed to help in the early diagnosis of COVID-19 based on entered symptoms or medical images such as computed tomography scans and algorithms such as deep convolutional neural networks [21]. These systems raise the hope of making medical diagnostics of COVID-19 and also other diseases faster, more efficient, and consistent, and thus more valuable as they are able to compare patient data with a database that is larger than any physician’s experience. Consequently, applying ML systems in patient care could make the difference between life and death by enabling more effective and efficient diagnostics [10,17].

### Challenges of Adopting Machine Learning Systems in Clinics

However, despite this enormous promise, the integration of ML systems also poses challenges that have prevented the widespread adoption of these systems in clinics to date [22]. More specifically, clinics cannot draw on their experience from adopting other HITs, as ML differs substantially from prior technologies. Specifically, ML systems learn from high volumes of data instead of being explicitly programmed [12]. Although traditional clinical decision support systems rely on rule-based systems that produce deterministic outputs, ML systems derive their solutions based on complex statistical methods, leading to several consequences. First, ML systems are becoming increasingly complex and commonly resemble black boxes; that is, their mechanisms for generating predictions are opaque to humans. For example, ML systems based on deep neural networks make predictions using millions of parameters, and humans cannot comprehend each and every calculation. Second, ML systems that learn from data will almost never be able to perform tasks perfectly, for example, make classifications with 100% accuracy [11,19]. This is mainly because of the ML system reliance on statistical patterns, which will never be able to cover all edge cases. Third, the operationalization of ML systems in practice is challenging, largely because complex relationships between different types of artifacts (eg, data sets, models, and source codes) have to be managed [23]. Whereas traditional clinical decision support systems rely on human-defined rules that are instantiated in software code, ML systems are a result of applying algorithms to data, thus creating an additional dependency. All artifacts have to be versioned, and their dependencies must be tracked to comply with regulations and ensure reproducibility. Owing to these complicating factors, organizations in various industries struggle to integrate ML systems into their processes. Therefore, initial research is looking at the challenges that ML systems pose in terms of organizational adoption [24-27]. However, clinics differ considerably from other organizations, as they not only possess unique structures, management processes, and requirements for HIT adoption but are also responsible for their patients’ lives [28]. In these medical settings, the characteristics of ML systems are particularly problematic as physicians and patients rely on profound diagnoses and the correct functionality of ML systems at any time [19]. Consistent with the call of Davison and Martinson [29] for more context-specific research, studies regarding the adoption of ML systems in clinics must, therefore, reflect on both, the specific characteristics of ML systems and clinics. Such context-specific research on the organizational adoption of ML systems in clinics is becoming more prevalent in recent times [10,30,31]. Thematically, researchers mainly investigate the individual acceptance of physicians [19,31] and the technical specifics of ML systems, such as their lack of transparency [32,33]. However, the problem with existing research is that most of these publications are merely reviews and rely on the personal understanding and experience of the authors. Rare exceptions are, for example, Hofmann et al [34], Sandhu et al [31], and Sun and Medaglia 35], who made use of qualitative research methods. Hofmann et al [34] examined the opportunities and challenges of ML systems in radiology, whereas Sandhu et al [31] and Sun and Medaglia [35] studied the introduction of 2 specific ML-based diagnostic decision support systems in clinics. Although these publications already offer a first insight into the possible factors along the adoption process of ML systems, they are not sufficient to understand the process in its entirety.

### Objectives and Research Approach

In particular, to our knowledge, no work exists that theoretically embeds the organizational adoption process of ML systems in clinics and presents it based on empirical evidence. Rather, current research focuses on individual acceptance criteria instead of taking a holistic, organizational perspective [19,31]. Therefore, clinics lack an integral overview of the requirements that ML systems imply and that they need to address to harness the potential of these systems for their diagnostic processes. Guided by the call of Shaw et al [10] for more research on the adoption of ML systems in clinics and the lack of prior integral research, our study thus aims to answer the following first research question: which specific factors influence the adoption process of ML systems in medical diagnostics?

Moreover, previous research does not elaborate on how these factors may manifest in a range of different stages and how these stages determine an overarching maturity score. However, such a maturity model could shed further light on the adoption process of ML systems in clinics by providing an empirically grounded and operationalized construct to measure adoption progress [36,37]. Therefore, the maturity model could not only be applied in future empirical research but also allow clinics to assess their as-is situation and evaluate potential courses of action for ML adoption. Therefore, our research sets out to investigate the following second research question: how can the identified factors be used to establish a maturity model for the adoption process of ML systems in clinics?

To answer these research questions, we conducted a qualitative study based on explorative interviews (N=22) with experts working for clinics or suppliers of clinics. To structure the key findings of our empirical investigation, we referred to the health care–specific framework of nonadoption, abandonment, scale-up, spread, and sustainability (NASSS) for a conceptual basis [38]. Although this adoption framework provides a foundation, it is not sufficient to represent the full adoption process of ML systems in clinics, given the particular characteristics of ML systems. To provide a more context-specific framework [29], we drew on qualitative data to gradually adapt and expand the existing framework by several factors specific to the adoption process of ML systems for clinical diagnostics. Moreover, we used qualitative data to develop a maturity model that can help researchers and clinicians understand the possible range of ML adoption stages in clinics and determine an overarching maturity score. Overall, we aim to provide a practical reference point for clinicians to integrate ML systems more effectively into their diagnostic processes.

In the next section, we describe our qualitative research design, introduce directed content analysis as our basic data analysis methodology, and explain the development process of the ML maturity model in detail. We then present the empirical results of our study to provide a valuable basis for further research and guidance to clinics aiming to integrate ML systems within their diagnostic processes. Finally, we conclude by discussing the theoretical and practical implications of our study and showing perspectives for future research.

## Methods

### Overview

**Figure 1:**
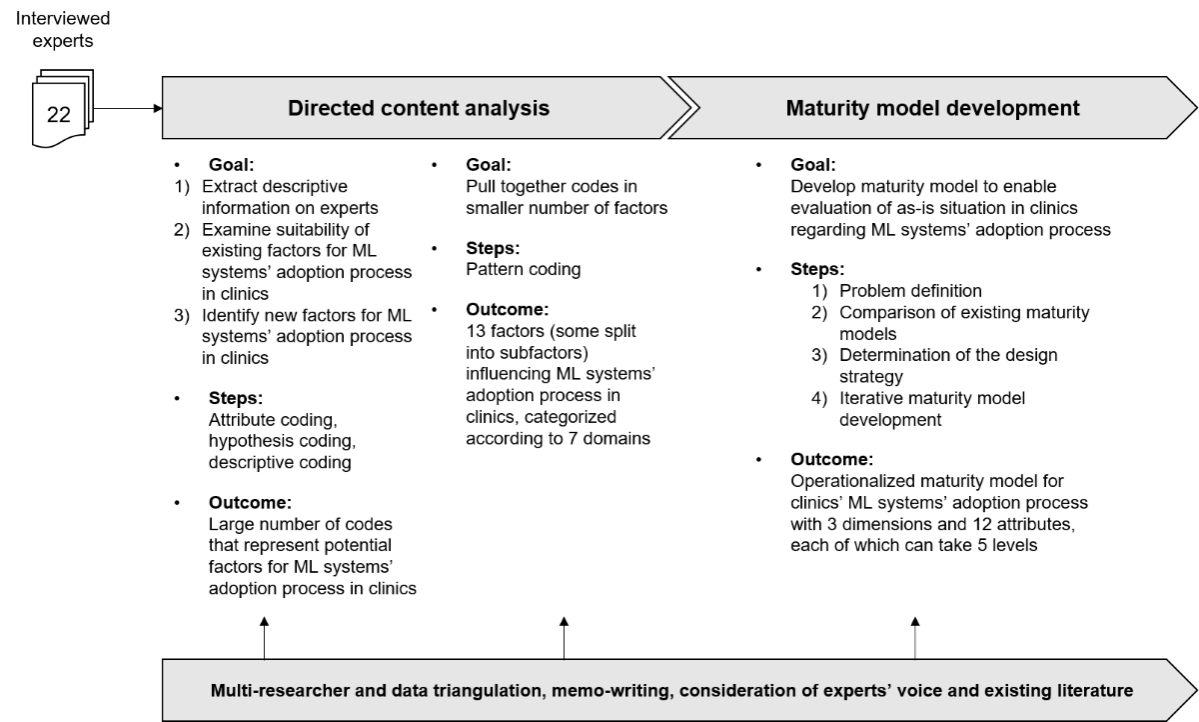
Overview of research procedure, illustration based on Jöhnk et al [25]. ML: machine learning.

Qualitative data provide a rich source of information that can help to better understand emerging, highly complex research subjects [39]. Therefore, to understand the complex adoption process of ML systems and derive a maturity model, we used a qualitative approach to “see the world through the eyes of the people being studied” [39]. In this regard, we applied the key informant method and conducted in-depth interviews with experts (N=22) who have particular qualifications and specialized knowledge on the topic investigated [40]. We led these interviews according to a semistructured interview guideline to ensure that all relevant questions were posed. The questionnaire included general questions about the person, questions about previous knowledge in the field of ML systems, the assessment of potentials and challenges of ML systems for medicine, and further, more detailed questions about the prerequisites in clinics to adopt ML systems for diagnostics. Owing to the qualitative approach, we kept the guideline open and flexible to allow adaptations to the respective interviewed expert, their position, and knowledge base [41]. We analyzed the qualitative data with the help of *directed content analysis* [42] and the methodological approach for *maturity model development* [36]. For an overview of the research procedure, please refer to Figure 1.

During the research process, we used several practices to obtain rigor and trustworthiness. To begin with, we defined 2 clear research questions and a conceptual framework that we used as input for our research design. Furthermore, we followed a theoretical sampling approach by iterating between data collection and analysis until we reached theoretical saturation [43]. In this way, we drew on the results from preceding interviews to select further experts and, for example, interviewed not only physicians and managers from clinics but also managers from HIT suppliers to obtain a more holistic perspective. In this regard, considering suppliers allowed us to gain an external, less biased perspective on the adoption of ML systems in clinics. Therefore, we found the additional supplier perspective to be particularly useful in triangulating the data and increasing the validity of our findings [44]. Moreover, different medical disciplines were considered in the interviews (eg, radiology, pathology, and internal medicine) to allow for different perspectives on medical diagnostic processes (eg, interpretation of medical scans, pathology slides, and electrocardiograms) and obtain more generalizable results [45]. The resulting number of interviews is comparable with those of other qualitative studies in IS health care research [31,34,46,47]. With regard to data analysis, we followed a structured and reproducible approach to evaluate the qualitative data [36,42]. During this whole process, a multiresearcher triangulation took place to include different perspectives on the research topic [44]. In that sense, we discussed all data analysis steps and results intensively with the authors and with further qualified researchers from the fields of IS, computer science, and medicine. We recorded the results of these discussions in the form of memos to make them available in the following analysis stages [48]. For later documentation of the results, we decided to include “the voice of participants” [49] and thus quote directly from the interviews while presenting our findings. Where possible, we have additionally incorporated existing—so far scattered—literature that backs up and contextualizes particular statements made by interviewed experts, thus demonstrating the relevance of the findings from the interviews [25].

### Data Collection and Sample Selection

Qualitative data were collected in 2 rounds. We conducted a first round of in-depth interviews from the second to the last quarter of 2019. This round of interviews included most participants (15/22, 68% of experts) and formed the basis for content analysis and maturity model development. However, the adoption of ML systems in clinics has progressed significantly in recent times. Therefore, we conducted a further round of interviews (7/22, 32% of experts) in the first quarter of 2021 to capture potential new insights from clinics on the research subject. Moreover, we shared the identified factors and the complete operationalized maturity model with the second-round interview participants to verify and refine the findings from the first panel. All the interviews were conducted in 2 European countries (Germany and Switzerland).

To identify suitable participants for both rounds of interviews, we searched for experts in professional networks, clinic websites, and at relevant conferences on ML in medicine. We interviewed qualified experts, who had detailed knowledge of clinical processes, had profound experience with ML systems, and were involved in the respective decision-making processes [50]. Of the 22 interviewed experts, 5 (23%) were physicians, 8 (36%) held a hybrid position (ie, physicians with additional leadership responsibilities), and 9 (41) worked as full-time managers or information technology staff in the medical field. The participants worked for 11 different clinics and 5 HIT suppliers. Four clinics are privately financed, and the others are public, providing a view of both privately and publicly funded clinics. All clinics and suppliers are currently running projects related to ML. On average, each expert interview lasted 48 minutes and took place in a private space. The interviews were audio recorded and transcribed after mutual agreement. In 3 interviews, we only took notes as the participants did not consent to recording. For an overview of the experts, see Table 1.

**Table 1:** Overview of interviewed experts.

ID			Position		Specialty		Expertise (years)
**Clinics:** **key informants of clinics**							
	C-01	Physician		Radiology		3	
	C-02	Physician		Radiology		15	
	C-03	Physician		Radiology		8	
	C-04	Physician		Cardiology		3	
	C-05	Physician		Neuroradiology		3	
	C-06	Physician^a^		Neuroradiology		9	
	C-07	Physician^a^		Internal medicine		19	
	C-08	Physician^a^		Internal medicine		35	
	C-09	Physician^a^		Pathology		18	
	C-10	Physician^a^		Radiology		37	
	C-11	Physician^a^		Gynecology		40	
	C-12	Physician^a^		Otolaryngology		25	
	C-13	Physician^a^		Cardiology		12	
	C-14	Chief technology officer		Cardiology		8	
	C-15	Chief technology officer		Biomedicine		20	
	C-16	Director		Internal medicine		12	
**Health information technology (HIT)** **suppliers:** **key informants of clinics’ HIT suppliers**							
	S-01	Director		Nephrology		20	
	S-02	Director		Biomedicine		22	
	S-03	Director		Genetics		10	
	S-04	Head of research and development		Radiology		2	
	S-05	System-engineer		Pathology		3	
	S-06	Innovation project lead		Surgery		3	

^a^Physician with leadership responsibilities.

### Directed Content Analysis

Our first goal was to identify the factors that are specific to the adoption process of ML systems in clinics and are not yet sufficiently covered by existing theories. As ML systems have an innovative character because of their novel, complex technical characteristics, we followed the steps of directed content analysis to extend existing theory on the adoption of innovations [42].

The process of adopting innovations in organizations is an overarching process that evolves from initial awareness of technology to a solidified interest and a subsequent adoption decision, to its implementation in the organization, and finally to continued adoption [51]. Presently, adoption research regarding HITs has started to look beyond the mere awareness of a technology to include the later stages of the adoption process [38]. In this context, ML systems own highly specific characteristics that will necessitate a significant change in the organization structure and working routines eventually [11,19]. Therefore, the whole adoption process of ML systems should be considered thoroughly. To capture this, we used the NASSS framework as a conceptual basis. NASSS has primarily been developed for the health care context by combining established health and social care frameworks and can be used to analyze the full adoption process of an HIT, including the implementation phase and continued adoption of the technology. It includes several *domains*, namely *technology* and its features, the *organization* that aims to adopt the *technology*, the *wider system* of an *organization*, the *condition* to be diagnosed and treated, the demand and supply side *value proposition* associated with HIT, and the *adopter system* consisting of patients, their relatives, and medical staff. Furthermore, it explicitly conceptualizes the *embedding* and *adaptation* of the HIT within a clinic over time [38]. Each domain, in turn, comprises several *factors* that specify the domain considered. These are, for example, the regulatory issues related to a technology (wider system) or the value a technology can have for a patient (value proposition). The suitability of the NASSS framework for the topic under study is evidenced by recent research calling for the use of the framework for empirical work on the adoption process of ML systems in clinics [30]. The NASSS framework forms the basis for our research but is insufficient to explain the specific adoption process of *ML systems* in clinics and, therefore, needs to be reconsidered. In this regard, we used the framework as a starting point, and it was adapted and expanded, taking into account the qualitative data [42].

Specifically, we applied an iterative multicycle coding process that is in line with directed content analysis, which consists of 2 coding cycles, between which we moved back and forth [52]. The first cycle comprised 3 different types of coding. Using *attribute coding* enabled us to receive descriptive information concerning the participant. *Hypothesis coding* was used to consider the prespecified conceptual framework (ie, NASSS) and to examine the suitability of existing domains and factors regarding the adoption process (eg, domain: value proposition; factor: patients’ value through ML). In contrast, the *descriptive coding* approach allowed us to identify new aspects that go beyond the conceptual framework by disregarding formerly identified domains and factors. As the coding procedure during the first cycle has led to a large number of constructs, we used *pattern coding* within the second coding cycle to pull together the codes into a smaller number of factors [52]. We performed the analysis using the NVivo 12 (QSR International) software. The result of the analysis is a holistic overview of domains, factors, and subfactors that influence the adoption process of ML systems for diagnostics (see section *Factors Influencing the Adoption Process of ML Systems in Clinics*).

### Maturity Model Development

In a further step of our data analysis, we aimed to use (a subset of) the factors identified during content analysis to create a maturity model that can help clinics to assess their current state in the ML system adoption process. Organizations can have different maturities with regard to the management of technologies. To determine the maturity score of an organization regarding a certain type of technology, specified maturity (assessment) models can be used [36]. These models constitute an instrument for organizations to “measure and assess domain capabilities at a given point in time” [53]. In this context, maturity models are valuable tools for organizations to assess and document their as-is state and, based on this, achieve directions for transformation and prioritization of potential investments [36,54]. Therefore, a maturity model comprises different *dimensions* that are subdivided according to specific *attributes*, each of which can take different *maturity levels*. Dimensions represent capability areas, for example, in the field of technology management, that should be exhaustive and distinct from each other. Attributes further specify these dimensions and represent practices, activities, or measures that can be taken by the organization and contribute to an organization’s maturity. Levels, on the other hand, are archetypal degrees of maturity which are often represented as a 5-step sequence of stages expressed by different labels [36,55-57]. Becker et al [36] differentiated 5 levels, namely, (1) *initial*, (2) *assessing*, (3) *determined*, (4) *managed*, and (5) *optimizing*. The descriptions characterizing these levels may vary depending on the level definitions and the subject of investigation. However, in general, an attribute is considered to be at an *initial* (1) level if the processes investigated are still in their infancy, chaotic, and not consciously controlled by the organization, whereas the most advanced level *optimized* (5) stands for those attributes whose processes are already actively and continuously improved with the help of standardized feedback mechanisms [55,58]. The overall maturity score of the organization, which can take one of the 5 levels described, results from the compilation of the individual attribute levels.

In recent years, maturity models have made their way into the health care sector. A literature review conducted by Carvalho et al [59] showed that clinical researchers and practitioners have established and applied various specified maturity models to understand and evaluate the integration of different HITs. However, there are no studies in the existing literature or insights from practice on a specific maturity model related to ML systems in clinics. To create a new maturity model for the ML adoption process in clinics, we followed the systematic development process outlined by Becker et al [36], which is loosely based on the design science methodology of Hevner et al [60]. This methodological approach includes 4 steps that structure the development of maturity models and 4 more that accompany the application of maturity models in practice. As our primary goal was to create a maturity model for the adoption process of ML systems in clinics rather than the subsequent application of the model in clinical practice, we focused primarily on the first 4 steps.

The first step of the maturity model development process by Becker et al [36] is to define the problem underlying maturity development. The aim of this study was to provide researchers and clinics with the opportunity to evaluate the clinic status quo in the adoption process of ML systems. As clinics still struggle to integrate ML systems into their processes, we consider this problem particularly relevant and topical [22]. After defining the problem domain and the target group, we searched for existing maturity models from adjacent research fields. In particular, we identified 3 maturity models that, although not specific to clinics, are drawn from the field of AI: the *artificial intelligence maturity model* by Alsheibani et al [61], the *five maturity levels of managing AI* by Lichtenthaler [62], and the *machine learning maturity framework* established by Akkiraju et al [63]. All of them use a 5-level maturity scale ranging from an *initial* (1) level to *optimized* or *integrated* (5). Although the framework by Akkiraju et al [63] was strongly technically oriented, Alsheibani et al [61] and Lichtenthaler [62] incorporated a management perspective as well. Although the identified maturity models helped provide a structure for the model to be built (eg, levels and potential attributes) and specific wordings that could be used (eg, “no data exist to train AI” [61]), no model is complete in itself or tailored to clinics. As clinics are highly specific in their structures and processes [28], we took initial ideas from the existing models but widely supplemented and concretized these ideas with the help of the content analysis results. In particular, we designed a new maturity model that is specific to ML adoption in clinics, but which incorporates some basic structures and descriptions from existing models. In the following core step, the actual development of the maturity model takes place. We adopted an iterative approach that included 4 substeps: design-level selection, approach selection, model design, and testing. In total, 3 iterations were performed to develop the maturity model. In the first iteration, the existing maturity models and the results of the directed content analysis were considered to build a basic concept. In the second iteration, additional researchers from the field of IS and computer science were brought in to discuss and optimize the maturity model. In the third round, the maturity model was shared, discussed, and tested with 8 of the medical experts [36]. Within these iterations, we decided to adopt a multidimensional maturity assessment based on the results of the previously conducted content analysis. In particular, a subset of 3 domains was used for the dimensions of the maturity model; the corresponding factors or subfactors form 12 attributes that further specify these dimensions. Thereby, only those domains and factors were selected that clinics can modify themselves and are not set by external forces that are beyond the clinics’ reach (eg, from the wider system). The resulting attributes were then populated with individual-level descriptions using the qualitative interview data. Therefore, we started with the 2 extreme levels *initial* (1) and *optimized* (5) for each attribute, and the formulations for the levels in between were derived from the interview data, the existing maturity models and literature, or logical inference. The complete maturity model, including dimensions, attributes, and levels, was then discussed with 8 of the medical experts, who confirmed its comprehensiveness, consistency, and adequacy. Following Joachim et al [64], the maturity model was mathematically operationalized to enable clinics to calculate an overall maturity score. In addition, we have developed a web application for using the maturity model that clinicians can apply to calculate their maturity level in the process of ML system adoption. The result of these iterative development steps is an evaluated applicable maturity model that can help researchers and clinics assess the current state of clinics in adopting ML systems (see section *A Maturity Model for ML Systems in Clinics*).

## Results

### Factors Influencing the Adoption Process of ML Systems in Clinics

#### Overview

**Figure 2:**
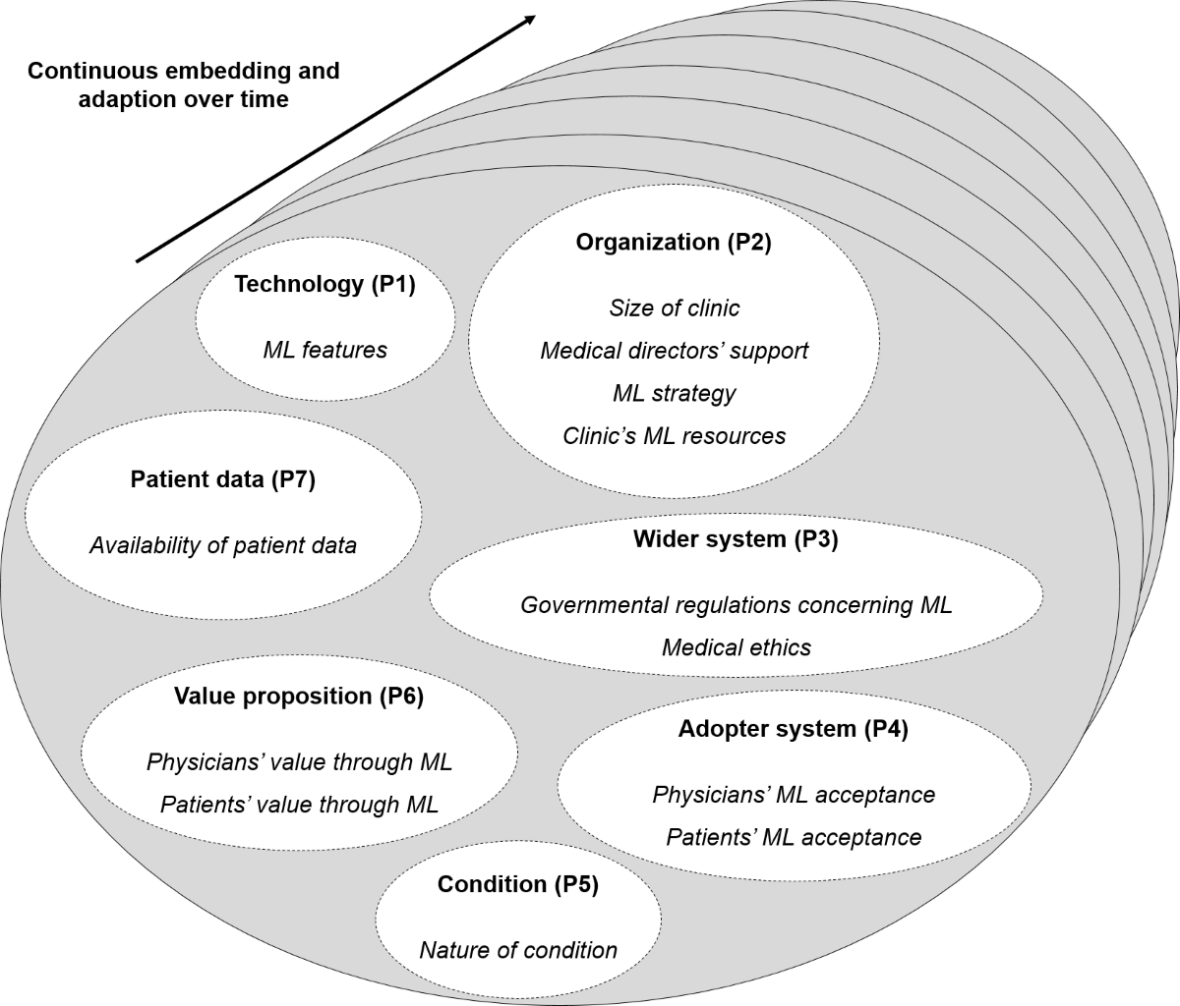
Integrative framework for the adoption process of machine learning systems in clinics. ML: machine learning.

As diagnostic procedures can differ within different medical specialties, the data analysis focuses on common factors that affect the adoption process of ML systems for diagnostics in clinics and can be derived across all disciplines. An integrative overview of these factors is shown in Figure 2. In the following section, we present and discuss the results of our directed content analysis. For this purpose, we structured our findings according to the domains: technology, organization, wider system, adopter system, condition, value proposition, and the new domain patient data. The aforementioned domains interact with each other to enable the continuous embedding and adaptation of ML systems in clinics over time [38,65,66]. In line with the existing literature, we thus did not formulate a separate domain to address the deep integration of ML systems across time. Rather, we assumed the embedding and adaptation over time to be a dynamic process in which, depending on the phase in the adoption process, specific domains and associated challenges are particularly relevant.

#### Technology

The features of technology are factors that are already considered within the original NASSS framework [38]. Nevertheless, as outlined earlier, ML systems encompass several highly specific characteristics that cannot be compared with those of other HITs. Therefore, the existing general technical features factor is not sufficient to capture the properties of ML and has to be specified further.

As one subfactor of *ML features*, the interviewees pointed out the *lack of transparency* of ML systems as a major obstacle for the clinic’s adoption of ML systems. ML systems based on neural networks can consist of multiple processing layers and up to billions of numerical weights, hampering the comprehensibility of ML systems to humans [11,32,33]. Especially in high-stakes decision-making processes such as medical diagnostics, this can lead to major issues, as ML systems do not always provide correct suggestions (S-05). As a result, the experts state that physicians need to know exactly what the critical features considered by ML systems are and how identified patterns lead to conclusions. This is required so that physicians can assess the ML system’s recommendations and suggest an appropriate diagnosis and therapy. One of the experts underlines this aspect:

You will never make these existential decisions dependent on a black box, where it is not possible to understand what led to the recommendation.

C-08

Another subfactor of ML features is the *ability to adapt* their functioning if being retrained on novel data. This can become relevant either when the ML system is transferred to another context (eg, another clinic) or needs to be retrained after some time; for example, new medical research results are gained or the patient demographic structure shifts. Clinics thus have to deal with an opaque system that is able to change its reasoning, making the outcome of an ML system unpredictable. Accordingly, experts see the adaptability of ML systems as another factor that has to be addressed by clinics (C-08, S-01, S-03, and S-05). To adopt ML systems, clinics need to have a clear strategy in place on how to cope with the opacity and adaptability of self-learning ML systems. Thus, we state our first proposition:

- P1: The features of ML systems (ie, lack of transparency and adaptability) will impede their adoption in clinics.

#### Organization

Looking at the organization domain, 4 factors emerged during the interviews. These are the *size of a clinic*, *medical directors’ ML support*, *ML strategy*, and *clinic’s resources for ML*.

The size of a clinic is a newly identified factor that was not specifically considered in the original NASSS framework. However, the interviewed experts emphasize that small clinics usually have fewer resources than large clinics, which could hamper the adoption of ML systems (C-15). In the specific context of ML systems, larger clinics further care for a higher number of different patients and thus have access to more patient data, which are needed to train ML systems appropriately (S-01).

Furthermore, experts state that clinic medical directors need to support the adoption of ML systems for diagnostic processes to guarantee financial and nonfinancial support for the new technology (C-03). In this regard, ML systems for medical diagnostics affect the core business of clinics and thus have strategic relevance [67]. As medical directors develop the clinic’s strategy, they are responsible for paving the way for the readiness of clinics to adopt ML systems. This is in line with prior research that states the significance of medical directors’ support regarding the adoption of strategically relevant HITs in clinics [68,69].

As ML systems are a strategically relevant innovation, not only is the support of the directors necessary but also the establishment of an overarching, long-term ML strategy. The importance of an innovation strategy is also confirmed by an expert who emphasizes its relevance, especially against the background of the adoption of ML systems in a hospital network:

When I want to launch it to the 1900 other hospitals, I have to think about a classic transformation strategy.

C-16

Such a strategy should include a plan of structured activities that contribute to the successful adoption of ML systems over time and should be supported by the clinic’s medical directors (C-03).

One of the most frequently stated factors within the domain organization is the clinic’s resource. This factor is similar to the factor capacity to innovate already included in the original NASSS framework but is subdivided into novel subfactors (ie, *clinic’s technical infrastructure*, *clinic’s financing structure*, and *clinic’s medical and ML methods expertise*). In line with existing literature [10,70], some of the experts report that clinics frequently rely on a wide range of clinical legacy systems, which are often proprietary to the suppliers, not connected, and based on outdated software and hardware:

The primary challenge [...] is that the clinic usually consists of [...] million proprietary systems that are not connected.

C-01

This difficulty is not only present within the clinic itself but also translates to the interorganizational level. Although some experts state that their clinics already have some special data networks in place, almost half of the experts stress that health care organizations have not yet connected their data to systems in and outside the clinic (C-01, C-03, C-04, C-05, C-06, C-08, C-09, C-13, C-15, and S-04). However, experts emphasize the importance of having a high-performance technical infrastructure that can efficiently access data from multiple sources, for example, via secure internal (within clinic) and external data networks (eg, clinic-to-primary care), which has the computing capacity needed to train ML systems (C-01, C-03, C-04, C-05, C-09, C-13, and S-04). Therefore, a clinic’s existing technical infrastructure could pose a major challenge to the adoption of ML systems.

Furthermore, the interviewed experts pointed out the problem of the current financing structure of clinics, which leads to strict budgetary constraints, especially in publicly funded institutions (C-04, C-05, C-11, C-12, and C-13). In this regard, an interviewee states that one part of their budget is assigned to daily costs, such as medication. The other part of the budget can be used to purchase large-scale medical equipment, such as x-ray systems. Thus, the development and setup of ML systems are not covered by either of the 2 parts, and often, no specific ML budget can be claimed (C-08).

Beyond that, there is a lack of personnel in clinics having expertise in both medicine and ML methods such as data science or data engineering:

The shortage of medical specialists hits us twice as hard. We feel this at the medical professional side [...], but it is also very apparent at the technical side.

C-14

Both fields of knowledge are regarded as highly important for the adoption of ML systems by many experts (C-01, C-04, C-05, C-14, and S-02). Although a medical background can help identify relevant training data or assess the functionality of the ML system, ML method expertise is needed to train, integrate, and operate ML systems as presently, only scattered out-of-the-box ML systems exist for application in medicine, requiring clinics to develop and maintain ML systems by themselves (C-01, C-14, and S-02). Therefore, clinics need specific expertise in the field of ML methods in addition to their medical understanding to develop, set up, and run ML systems in clinics. In sum, we propose the following:

- P2: A larger clinic size, medical directors’ ML support, formulation of an ML strategy, and availability of resources for ML (ie, technical infrastructure, ML budget, expertise in the field of medicine, and ML methods) will facilitate the adoption of ML systems in clinics.

#### Wider System

With regard to the wider system, there are 2 relevant factors influencing the adoption of ML systems: *governmental regulations concerning ML* and *medical ethics*. Governmental regulations are a factor already known from the original NASSS framework. Nevertheless, the interviews revealed some particularities that were not covered by the general concept and are described below. Medical ethics is a factor that has not been captured by the NASSS so far but has been identified through our study.

In the field of medicine, there are several governmental regulations that must be taken into consideration when adopting ML systems. The following subfactors could be identified: *medical approval of ML systems*, *accountability*, and the *protection of sensitive personal data*.

The experts drew attention to the fact that HIT offered in the market and used in clinics is subject to several laws. This includes the need for medical approval conducted by legal authorities or HIT suppliers themselves (C-03, C-05, and C-12). In the United States, the Food and Drug Administration is responsible for the admission of medical products. In Europe, the HIT suppliers themselves need to perform a conformity assessment procedure, for example, based on the Medical Device Regulation [71,72]. As mentioned before, most ML systems are currently being developed by the clinics themselves and have not undergone any approval process (C-03). However, legal approval of ML systems is not trivial, as the systems can learn from new experiences and adapt themselves as described above:

It is not obvious how evidence can be obtained for an [ML] model that differs significantly at the beginning, middle, and end of the study. If you want to approve a medical device today, you have to describe the intended use in detail.

S-01

The Food and Drug Administration addresses this legal uncertainty in an official statement that proposes an action plan for innovative approaches to more effectively approve adaptive ML systems [72]. The European Medicines Agency is also still in the early stages of defining and establishing an approval process for ML systems [73]. Therefore, legal ambiguities could represent a hurdle for clinics to adopt ML systems for diagnostics.

In addition to the medical approval of an ML system, there is the question of accountability for diagnoses. The experts interviewed indicated that it is questionable who takes over responsibility if the diagnosis prepared by an ML system is inaccurate (C-06, C-14, and S-05). It is also unclear who can be held liable—the HIT provider, the clinic, or the physician who is providing the medical diagnosis. An expert underlines this aspect with the following words:

Then there are certainly [...] legal problems, for example: who is responsible for the interpretation and possibly wrong results of the ML model?

C-14

According to the current state of the art, ML systems cannot be held responsible for their output, as a registered physician is always obliged to validate and interpret the system’s results and perform the final diagnosis (C-16). However, it would ease the decision of clinics to opt for ML systems if there were a legal specification, especially if ML systems are increasingly able to automate steps of sensitive processes such as diagnostics (C-14 and C-15).

Another subfactor of governmental regulations, which could be identified as relevant for the adoption process of ML systems for diagnostics, is the protection of sensitive personal patient data. Patient data are widely considered as highly sensitive [74] and are under special protection by national and international laws (C-02, C-04, C-13, S-02, and S-05). For example, the General Data Protection Regulation in Europe only permits the processing of health data if the patient explicitly accepts or if the clinic can provide particular reasons for the use of the data [75]. Thus, the respondents emphasized the clinics’ concerns in obtaining the necessary patient data to train the ML system (C-02, C-10, and S-06).

Using ML systems for diagnostic processes fueled medical ethics concerns among interviewees. On the one hand, ML systems are able to improve the efficiency and effectiveness of diagnostics (C-15, C-16, and S-02) and, on the other hand, the suggestions provided by ML systems are deduced based on statistical methods that recognize patterns in patient data that can be biased (C-15). Furthermore, the experts claimed that ML systems that are fed with patient data could determine whether a patient tends to develop a disease. This type of medical application would contradict the “patient's right not to know” (C-15). Summarizing these remarks, we set up the proposition:

- P3: Uncertainties in governmental regulations, strict requirements for the protection of sensitive patient data, and existing medical ethics will impede the adoption of ML systems in clinics.

#### Adopter System

The NASSS framework suggests that the successful adoption of ML systems is strongly influenced by individuals who are supposed to use the system or are affected by their suggestions. In this context, 2 ML-specific factors turned out to be relevant according to the interviews, which further specify the domain: *physician* and *patient ML acceptance*.

More than half of the interviewed experts stated that physicians’ acceptance is essential for the adoption of ML systems in clinics (C-01, C-02, C-03, C-05, C-06, C-08, C-09, C-12, C-14, C-15, S-03, and S-06). As ML systems have the ability to solve tasks that were previously performed by humans, physicians might feel interchangeable in their job (C-03, C-07, S-03, and S-05). ML systems are trained on large sets of data that exceed the experience of any single physician, setting new standards for medical diagnostics. In this regard, most experts are concerned that physicians could reject ML systems for their daily work:

As a doctor who may have ten or 20 years of experience [...], would I like to be taught by a machine [...]?

S-03

These concerns have recently found their way into pertinent research, demonstrating the relevance of the topic [19,30,31,34]. However, it is also evident that the acceptance of ML systems differs among different age groups. In particular, physicians who belong to the group of digital natives are more willing to understand and ultimately use ML systems (S-04 and S-06).

Most interviewees stated the importance of patients’ views on the use of ML systems for medical diagnostics. Although a physician is still involved in the decision-making process, patients might refuse the use of an ML system as the physician may be influenced by suggestions for possible conditions that are derived statistically and could be affected by biases. Furthermore, personal, sensitive patient data have to be processed to gain results. Therefore, experts state that patient acceptance of ML systems is highly relevant for the adoption of ML systems for diagnostics (C-02, C-06, and C-14). We thus conclude the following:

- P4: Physicians’ and patients’ acceptance of ML systems will facilitate the adoption of ML systems in clinics.

#### Condition

As specified within the NASSS framework, patient condition affects the applicability of a technology. This is not only the case for conventional HITs but also holds true for ML systems, as stated by the interviewed experts (C-02 and C-09). ML systems have a narrow focus and can only deal with specific delimited problems [11,12]. However, the human body is a highly complex and not fully understood system that can hardly be delineated. Medical conditions can be complex, poorly understood, or even unpredictable, for example, when multiple comorbidities are involved, making it difficult for ML systems to provide a clear diagnostic recommendation (C-02 and S-02). Therefore, the nature of the condition affects the applicability of ML systems, which can only handle delimited problems in the diagnostic process. Thus, the use of ML systems will be limited to the diagnosis of certain conditions:

- P5: The limited applicability of ML systems for the diagnosis of specific conditions will impede the adoption of ML systems in clinics.

#### Value Proposition

The value proposition is another domain of the NASSS framework that we were able to concretize by analyzing the interviews. According to the experts, the adoption of ML systems could result in the creation of *value for* both *physicians* and *patients* (C-03, C-10, and C-14).

Integrating ML systems in their daily work enables physicians to improve the effectiveness and efficiency of their diagnostics as they can base their decisions on a broad database that is evaluated within a few seconds (C-16):

If you have the choice among a pathologist who has already looked at 10,000 cuts [...] compared to one who has created only 500 findings, whom would you chose? But [...] AI has not only 10,000 but 500,000 findings in its memory.

C-08

In this regard, ML systems that are, for example, based on image recognition algorithms can surpass the ability of the human eye to capture details and patterns in x-rays [76]. If used for a second opinion, ML systems thus increase the quality of physicians’ work (C-02 and C-11).

In addition, patients could directly benefit from a decision that is faster and more informed if physicians use ML systems for diagnostics as a supportive tool (C-10 and C-16). We thus propose the following:

- P6: The additional value for physicians and patients created through ML systems will facilitate the adoption of ML systems in clinics.

#### Patient Data

During the interviews, nearly all experts stated the *availability of patient data* as crucial for the adoption of ML systems for diagnostics. In this regard, patient data have to be available to develop and train the ML system in the first place and subsequently retrain it during use. This factor comprises various subfactors (ie, *digitization of patient data*, *unified data formats*, *data quality standards*, *data anonymization*, and *representativeness of training data*) which are described in the following section.

According to the experts, most clinics generate high volumes of patient data through their daily diagnostic processes (C-03, C-05, S-01, S-04, S-05, and S-06), which is basically a positive feature as an appropriate amount of data is needed to train ML systems [11,20,35,77]. However, although high volumes of data are generated, many processes in clinics are still paper-based, which lowers the proportion of patient data available in digitized form:

Data are often not digitized, much is still in paper files, not structured, which means that the data availability is really extremely [...] poor.

C-03

This observation is in line with prior research concerning clinics that are lagging behind at using digitized technologies and digitizing patient data [1]. As a consequence, the interviewed experts see the integration of an electronic medical record system as a prerequisite for the application of ML systems (C-16, C-03, C-04, and C-13).

Furthermore, interviewing the experts revealed that medical patient data, if available in digitized form, are usually provided in a variety of proprietary data formats as many disparate clinical legacy systems from different suppliers have to interact to enable physicians to provide laboratory tests, diagnostic images, or clinical notes. These proprietary data formats are often difficult or impossible to convert, making the generation of consistent formats highly problematic (C-03, C-04, and S-04). The problem of differing data formats in clinics has already been recognized outside the ML context, for example, in research on the adoption of cloud solutions in health care environments [78]. Nevertheless, it is particularly critical for the introduction and use of ML systems that the patient data be processed for training and retraining the system. Although the first research has been conducted to allow for the transformation of different medical data types in one format [79], most clinics have not yet been able to implement unified standards for patient data to enable processing and analysis by ML systems.

Furthermore, digitized patient data are often stored in unstructured file types, such as images, texts, or videos (C-01, C-03, C-07, C-13, C-15, and S-04). The experts cautioned that the quality of unstructured data is highly dependent on the particular clinic where the data are generated and their clinical staff (C-06, C-07, and S-04). For instance, physician letters are frequently written in free text formats, which are filled with synonyms and can be interpreted individually. More specifically, personal formulations are used, such as the description of a tumor size as compared with that of a walnut (C-07). Thus, patient data are not only hard to harness and have to be transferred to a machine-readable format first (C-03 and C-04) but also lack common quality standards (S-04), impeding the extraction of generalizable patterns through ML. Clinics aiming to adopt ML systems to support their diagnostics should therefore set standards for data creation, for example, by establishing a common language that physicians use when creating free texts. Such efforts are already being driven by some in-clinic as well as national initiatives (C-12 and C-16). In addition, other primary structured data sources could be connected, such as data from laboratory findings, to complement the unstructured data [80].

Moreover, the experts strongly emphasize that clinics that want to use patient data to train ML systems need to anonymize the sensitive data before processing them through an ML system (C-15 and S-06). However, anonymizing data might remove valuable information, which could be important for obtaining a diagnosis. For instance, information about a person's residence could facilitate a diagnosis if a disease is more prevalent regionally (C-15). Therefore, it is necessary for clinics to find the right balance of anonymization and information value to be able to use the data despite data protection regulations and still preserve all the information necessary to find meaningful correlations through ML systems. The first steps are already being taken in technical research to balance protection and the quality of sensitive data effectively [81,82].

According to the experts, the selection of the right training data is especially important in a health care context, as wrong diagnoses may have an impact on patients’ lives. This leads to another aspect of patient data to be considered: the representativeness of training data. Patients in clinics vary in many aspects, from an outer perspective (eg, age, gender, and hair color) as well as from inner functioning (eg, size of organs and blood values; C-01 and S-05). If ML systems are trained based on an external database (eg, collected via data exchange) that is demographically or regionally skewed compared with the clinic’s conditions, false conclusions could be drawn by the system. In this context, an expert raised the example of an ML system supporting the detection of skin melanomas, which is mainly trained on a sample of patients with a similar phenotype. Therefore, this pretrained ML system cannot be easily transferred to patients of other ages or with other skin pigmentations (C-01). In addition, the representativeness of the data is affected when different clinical systems, such as different radiographic systems, collect data as the resolution of the medical equipment may vary from provider to provider (S-04). As training data for supervised learning need to be labeled by humans, the same could be said regarding the expertise and working philosophy of physicians, which could be highly heterogeneous depending on the physician’s knowledge state and working environment (C-09, C-14, and S-05).

The availability of patient data is a factor that is decisive for the adoption process of ML systems that need to be fed and retrained:

- P7: The availability and exchange of a large amount of digitized patient data for training (that are uniformly formatted, of high quality, anonymized but informative, and representative of the clinic) will facilitate the adoption of ML systems in clinics.

### A Maturity Model for ML Systems in Clinics

#### Overview

Against the background that no maturity model for the adoption process of ML systems in clinics could be found in research and practice, we created a concept for a maturity model and present the model below. On the basis of our empirical results, the model is intended to enable researchers and clinics to quantify the overall maturity of clinics within the adoption process of ML systems. We followed the design process of Becker et al [36] to conceptualize a maturity model that comprises 3 dimensions and 12 attributes, each of which is operationalized by 5 corresponding levels (Multimedia Appendix 1). The dimensions and attributes are derived from a subset of the results presented in the previous section, whereby the dimensions were inferred from the domains and the attributes from the factors or subfactors that can be modified by the respective clinic itself. Specifically, the dimensions organization (P2), adopter system (P4), and patient data (P7) and their respective subfactors were taken into account, as these can be controlled by the clinic itself, whereas the technology (P1), the wider system (P3), the condition (P5), and the value proposition (P6) are influenced by factors that are not in the hands of a single organization.

It is necessary to operationalize the model mathematically to render the maturity model applicable for research and practice. To this end, we followed the approach of Joachim et al [64], which has already been used for the operationalization of other maturity models (eg, in the area of business intelligence [83]). We assume that maturity evolves linearly in 5 levels *l*∈ *L* with L={1,2,3,4,5}, starting with *initial* (1) and ending with *optimized* (5) [83]. The maturity model for the adoption of ML systems in clinics consists of 3 dimensions, *d*, each of which consists of a set of attributes *I_d_* in turn. Therefore, the overall maturity score of a clinic is composed of the maturity score of all dimensions, whereby the maturity of each dimension *d* depends on the maturity within the corresponding attributes *a* ∈ *I_d_*. As a clinic can have different maturities in the different dimensions and attributes of a dimension, a stepwise estimation of the overall maturity score must be made. Therefore, a two-step process is followed in which (1) the *maturity score of the dimensions* (ie, *Mat_a_*) is determined first based on the respective attributes, followed by (2) the calculation of the *overall maturity score of a clinic* (ie, *Mat*).

#### Maturity Score of the Dimensions

At the lowest layer, each attribute *a* can take a value *x_a_* ∈ *A* with A={1,2,3,4,5} depending on the actual maturity of the clinic regarding the attribute, ranging from initial (1) to optimized (5). To determine the actual maturity value of each attribute in a dimension, a clinic must assess its own as-is situation by comparing the level descriptions (within each attribute) with their current adoption state in the clinic (Multimedia Appendix 1). For example, a clinic has a maturity value of *x_a_*=1 for the attribute *digitization of patient data* if it has nearly no digitized data available for training ML systems and is thus at an initial level of maturity. In the next step, all maturity values *x_a_* of the attributes within a dimension *d* are compared with all possible maturity levels *l* to determine the level with the smallest distance to the set of attributes of a dimension. To operationalize the comparison, a weighted Euclidean distance metric *Dist_d_(l)* is used in line with prior research [64,83]:



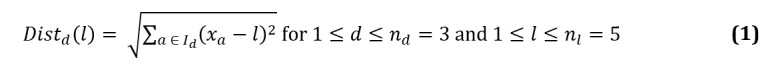



where *n_d_* represents the total number of dimensions and *n_l_* is the total number of levels. As a result, each clinic receives 5 distance values (for 5 levels, *l*) per dimension. To obtain the maturity score of a dimension *Mat_d_*, the level *m* associated with the minimum of these distance values needs to be selected per dimension:



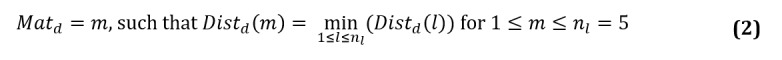



#### Overall Maturity Score of the Clinic

On the basis of the distinct maturity scores *Mat_d_* of the 3 dimensions, the overall maturity score *Mat* can be calculated in the second step. Again, we use a Euclidean distance metric *Dist(l)* to compare the maturity scores of the dimensions with levels *l* (Equation 3). The final overall maturity score of a clinic striving to adopt ML systems is determined by the minimum distance (Equation 4):



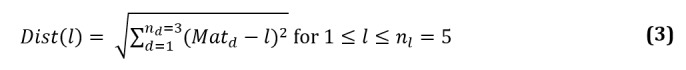





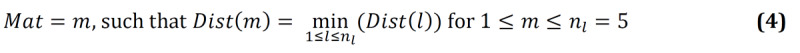



**Figure 3:**
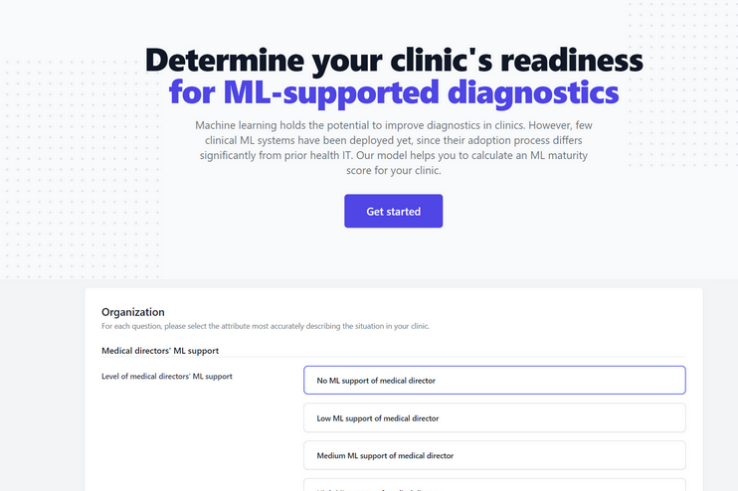
Determine your clinic's readiness for machine learning–supported diagnostics (screenshot 1 of the web application). ML: machine learning.

**Figure 4:**
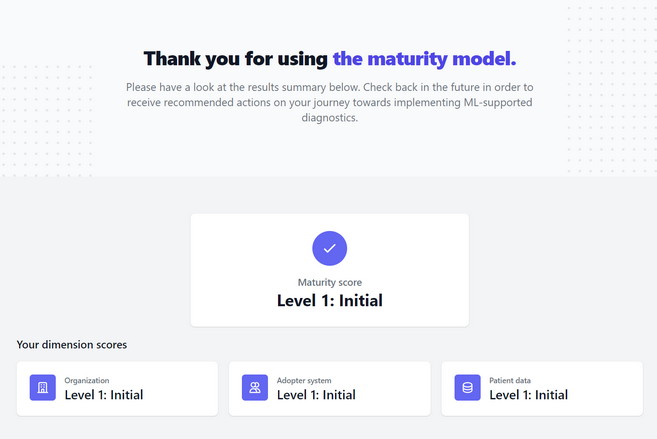
Thank you for using the maturity model (screenshot 2 of the web application). ML: machine learning.

To make the maturity model easily applicable for practitioners from clinics and researchers in the field of adoption science, we have developed a free-access web application based on the described mathematical operationalization, which calculates the maturity level of a clinic based on a questionnaire (Figures 3 and 4). This questionnaire includes the attributes as well as their level descriptions and is provided on the web [84].

## Discussion

### Principal Findings

ML has an impact on all areas of human life, including the health care system. In this regard, ML systems offer the opportunity to make diagnostics more efficient and informed. However, to harness ML for such an application, clinics need to deeply integrate ML systems into their clinical practice, a challenge that most clinics have not yet been able to overcome [20]. As clinics own highly individual, patient-oriented processes, it is crucial for researchers to reflect on this specific context [28,29]. However, prior research is lagging behind to provide empirically proven factors that influence the adoption process of ML systems in clinics for diagnostic processes. To address this shortcoming, we set up a qualitative study to (1) establish an integrated overview of factors specific to an ML system adoption process in clinics based on the NASSS framework and (2) create an operationalized maturity model that clinics can apply to assess their as-is state of ML adoption progress to decide on further actions and prioritize investments.

### Limitations and Future Research Opportunities

Before we discuss our contributions to theory and practice in detail, it is necessary to clarify the limitations of this study and show room for further research. As we pursued a qualitative approach, our results are based on the expertise of the 22 interviewees. To counteract potential problems of generalizability, we have not only applied various criteria to ensure rigor and trustworthiness of our study (eg, theoretical saturation, multiresearcher and data triangulation, and inclusion of multiple medical disciplines) but also carefully selected only highly involved experts. Nevertheless, it might be interesting for further research to perform a follow-up study to validate the proposed framework and maturity model quantitatively. In this regard, it might be informative to evaluate the derived maturity model by applying it in clinics. In doing so, it could also be investigated whether practitioners attach different importance to attributes and dimensions. On the basis of these findings, the maturity calculation could be adjusted by introducing weights for attributes and dimensions.

Moreover, we conducted the interviews in only 2 European countries. As health care systems vary across nations, interviewing experts from other regions with different economic and cultural prerequisites could lead to differing results. Nevertheless, the relevance of the findings for the international context was substantiated with the help of existing literature and practice contributions from international authorities, which are cited in the *Results* section. For example, the report of the Food and Drug Administration shows that the issue of medical approval of ML systems is also being discussed in the United States [72]. However, replication of this study in other countries would be useful to highlight possible differences within the adoption process of ML systems in clinics.

In addition, the rapid development of increasingly advanced ML algorithms could lead to systems that can not only augment but also automate diagnostic processes. Investigating automated diagnostics, which has not yet been applied in clinics, could produce different findings, although the results obtained in this study could provide first indications.

### Theoretical Contributions

Despite the limitations discussed, our study makes several important contributions to research. To begin with, we demonstrated that the NASSS framework can be applied but has to be adapted and expanded to explain the full adoption process of ML systems for diagnostics in clinics. To the best of our knowledge, this is the first study to provide an empirically proven and integrative overview of the factors determining the adoption of ML systems for clinical diagnostics and thus show what clinics need to consider to effectively integrate ML systems into their processes. Therefore, we contribute to and extend prior adoption research in health informatics, which has recently called for looking at the entire adoption process of HITs rather than just the initial awareness of the technology [38]. Although the identified factors are specific to diagnostic processes, it is conceivable that they may be applicable to other scenarios in which the cost of errors is high, such as ML-based treatment recommendations or medical prognoses in clinics.

Moreover, we have developed the first maturity model for ML system adoption in clinics, which contributes to the IS and medical body of knowledge by providing an empirically grounded and strategically derived artifact that depicts medical and ML-specific attributes and their level descriptions in detail. More specifically, the maturity model shows which attributes determine the status quo of clinics in adopting ML systems, how these attributes may manifest in descriptors according to 5 different maturity levels, and how clinics can evaluate their as-is state in the adoption process of ML systems. Researchers can apply the developed maturity model, for example, as an instrument in statistical studies investigating the adoption of ML systems in clinics. More specifically, the model can be used to operationalize the dependent variable in structural equation models or as a variable for multigroup comparisons [85], for example, to study the antecedents of clinical adoption of ML systems. Therefore, both the adoption framework and the maturity model for ML systems in clinics can guide future health care–centric research that seeks to explore the promises and challenges associated with ML systems in a medical setting.

### Practical Contributions

In addition, the empirically based results hold relevant findings for practitioners, who are increasingly facing rising health care costs, demographic changes, and overcrowding of the clinics, and thus need to improve the efficiency and effectiveness of their clinical processes. ML systems could be a solution to these problems but have so far only been sporadically integrated into clinics [22]. In fact, our qualitative study shows that most clinics still have major problems integrating ML systems into their diagnostics. In this regard, the derived framework provides medical directors with a holistic overview of potential enablers and inhibitors during the adoption process of ML systems in clinics and could provide a roadmap for practitioners.

Moreover, the developed maturity model can be used by clinics to obtain the first impression of their as-is situation in the adoption process of ML systems and to quantify it in an overall maturity score (see the website [84] to easily apply the model). Assessing the maturity score with the help of the model not only helps to make external comparisons between clinics but also to identify internal deviations of certain attributes from the overall status. This allows clinics to invest especially in these attributes that are far from the present overall performance and lower the clinic’s maturity score significantly to date. Thereby, the maturity model allows practitioners working for clinics to analyze their clinic’s current status quo, identify shortcomings, prioritize possible courses of action, and efficiently allocate scarce resources depending on the respective degree of maturity. In this way, our research can help practitioners identify tailored requirements for the successful adoption of ML systems in clinics and build relevant capabilities and resources needed in the age of AI.

## Appendix

Multimedia Appendix 1 Maturity model for machine learning systems in clinics.

## Abbreviations

AI: artificial intelligence
HIT: health information technology
IS: information systems
ML: machine learning
NASSS: nonadoption, abandonment, scale-up, spread, and sustainability
