# Supplementary figures and images for: Adoption of Machine Learning Systems for Medical Diagnostics in Clinics: Qualitative Interview Study
Source: J Med Internet Res. 2021 Oct 15;23(10):e29301. doi: 10.2196/29301 (PMC8556641; doi:10.2196/29301)

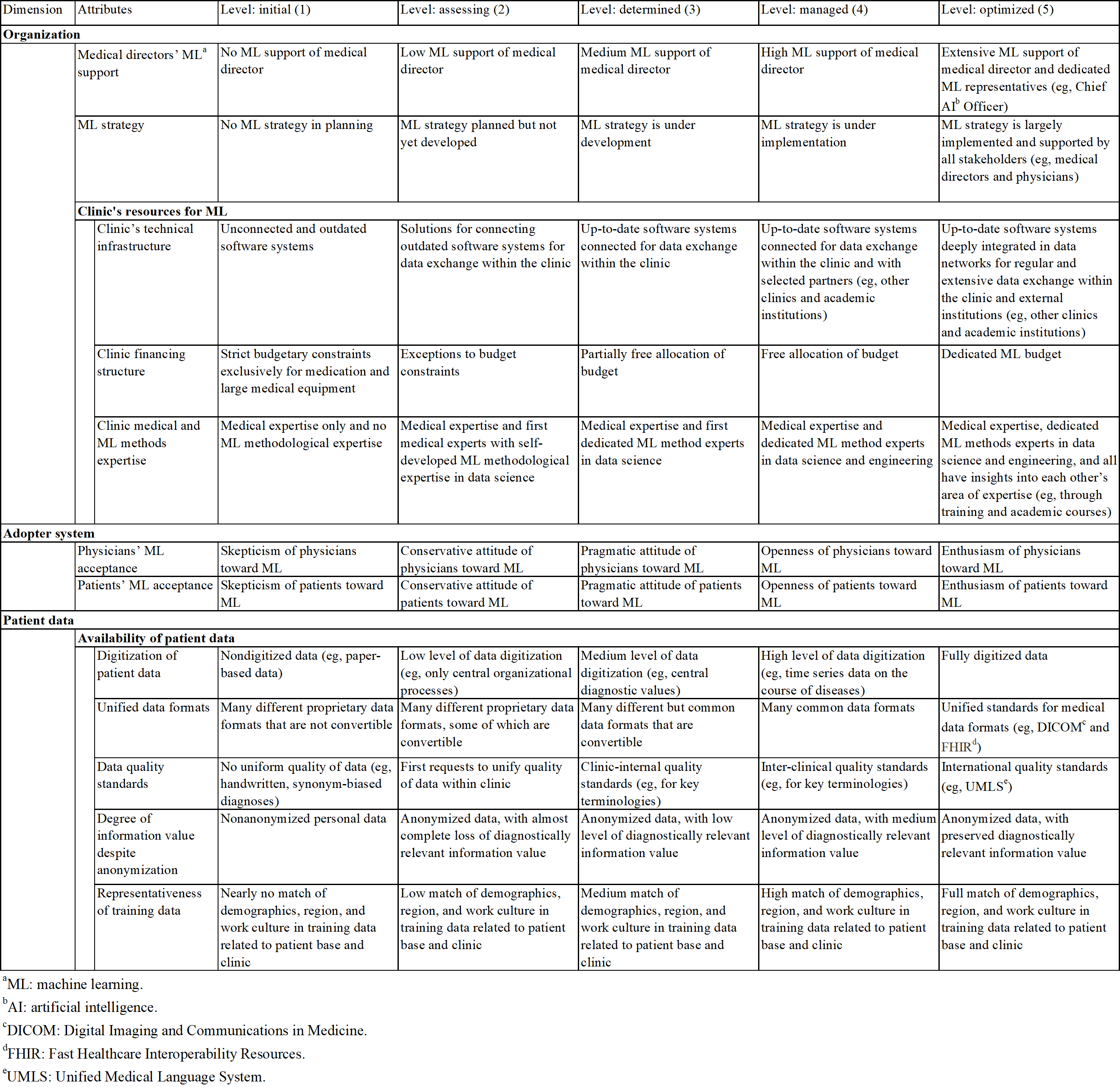

Supplement: Multimedia Appendix 1 [file jmir_v23i10e29301_app1.png]
